# Heavy Metal in Rice and Vegetable and Human Exposure near a Large Pb/Zn Smelter in Central China

**DOI:** 10.3390/ijerph182312631

**Published:** 2021-11-30

**Authors:** Yanxin Hu, Chuan Wang, Zhengcheng Song, Min Chen, Li Ding, Xingyu Liang, Xiangyang Bi, Zhonggen Li, Ping Li, Wei Zheng

**Affiliations:** 1 State Key Laboratory of Environmental Geochemistry, Institute of Geochemistry, Chinese Academy of Sciences, Guiyang 550081, China; huyanxin1102@126.com (Y.H.); wangchuan@mail.gyig.ac.cn (C.W.); zhengcheng_song@163.com (Z.S.); liangxy98@163.com (X.L.); 2 College of Earth and Planetary Sciences, University of Chinese Academy of Sciences, Beijing 100049, China; 3 School of Public Health, Guizhou Medical University, Guiyang 550025, China; chenmin3991@163.com (M.C.); dinglibright@163.com (L.D.); 4 Hubei Key Laboratory of Critical Zone Evolution, School of Earth Sciences, China University of Geosciences, Wuhan 430074, China; bixy@cug.edu.cn; 5 School of Resources and Environment, Zunyi Normal College, Zunyi 563006, China; lizhonggencn@126.com; 6 Institute of Atmospheric Environment, Chinese Academy of Environmental Planning, Beijing 100012, China

**Keywords:** Pb/Zn smelter, Pb isotopes, heavy metals, health risk

## Abstract

Non-ferrous metal smelting is a significant source of anthropogenic heavy metal emission and has led to severe environmental pollution that ultimately threatens the health of local residents. In this study, we determined concentrations of copper (Cu), zinc (Zn), cadmium (Cd) and lead (Pb), as well as Pb isotopic compositions in rice, vegetables and human hair in areas surrounding the Zhuzhou Pb/Zn smelter in Hunan, China and we assessed the health risks associated with rice and vegetable consumption for local residents. Results showed that rice and vegetable samples were significantly contaminated by Cd and Pb. Age and source of rice were important factors for the enrichment of heavy metal concentrations in human hair. The ratios of Pb isotopes in human hair (1.164–1.170 for ^206^Pb/^207^Pb and 2.102–2.110 for^208^Pb/^206^Pb) were comparable to those in rice (1.162–1.172 for ^206^Pb/^207^Pb and 2.098–2.114 for^208^Pb/^206^Pb) and were slightly lower than those in vegetables (1.168–1.172 for ^206^Pb/^207^Pb and 2.109–2.111 for^208^Pb/^206^Pb), indicating that Pb in human hair mainly originated from food ingestion. A non-carcinogenic risk assessment showed that Cd exposure was the dominant health risk for local residents. This study suggested that crops planted surrounding the smelter were seriously contaminated with Cd and human exposure was related to dietary intake.

## 1. Introduction

Non-ferrous metal smelting was one of the most important sources of heavy metal emissions and heavy metals such as copper (Cu), zinc (Zn), cadmium (Cd) and lead (Pb) were discharged to the surrounding environment during the Pb and Zn smelting process, causing adverse effects on the environment and human health [1,2,3,4,5,6,7]. Heavy metals in soil can be absorbed by plants [8] and enter human body through dietary intake [9]. Studies have shown that food consumption has been the main pathway of heavy metals exposure for humans in polluted areas apart from occupational exposure [10]. Cu and Zn are essential for the human physiological function of the human body at appropriate concentrations, while a deficit or excess of Cu and Zn can cause several diseases [11,12]. Cd and Pb are non-essential elements for the human body and can adversely affect human health at high levels of exposure. Cd is a toxic heavy metal and is classified as a Group 1 of human carcinogens [13]. Long-term Cd exposure can lead to chronic and acute adverse health conditions in humans, including kidney damage, osteoporosis and lung cancer [14,15,16]. Excessive Pb in the human body may cause renal dysfunction, damage to the central nervous system and a decrease in intelligence quotients (IQ) in children [17].

Hunan Province is a famous non-ferrous metal smelting center in China and the smelting industry is a foundational industry in this region [2]. In addition, Hunan is a major grain crop producer and rice production in this province ranked second in China in 2018 [18]. Therefore, food safety is particularly important. Studies have demonstrated that soils surrounding a non-ferrous metal mining and smelting plant were severely impacted by waste emission containing heavy metal [1,19]. Rice and vegetables grown near the smelting areas were severely polluted by heavy metals, especially for Cd [2]. In addition, intake of contaminated water, meat and eggs would also be subject to health risks for residents [4]. However, the concentrations of heavy metals in different sources and types of food are different, which leads to uncertainty of the health risk for different people. Few studies have investigated human heavy metal exposure and associated health risks through different types of food consumption in local residents.

Biomarkers are considered an important tool for estimating levels of human exposure to environmental pollutants. Human hair, a more stable matrix than urine and blood [20,21], has been widely used to assess human heavy metal exposure due to the higher feasibility of collection and storage than that of urine and blood [22,23,24]. Reference values of Cu, Zn, Cd and Pb in hair for adults given by the Trace Element Research Council of China (TERCC) are 8.0–20.0, 120–210, <0.6 and <10 mg/kg, respectively [25]. Moreover, due to the stability during physical and chemical processes [26], Pb isotopes have been widely used to trace the sources of Pb contaminations in soils, sediments, vegetables and biomarkers [27,28,29,30].

In this study, we aim to assess the heavy metal contamination in rice and vegetables grown in areas surrounding a Pb/Zn smelter, and the human exposure and health risks through dietary ingestion. Concentrations of heavy metals (Cu, Zn, Cd and Pb) in human hair were also investigated. The main impact factors of heavy metals in human hair were analyzed and the Pb isotope approach was utilized to trace the sources of Pb pollution in human hair. Potential non-carcinogenic risks for rice and vegetable ingestion were also calculated by target hazard quotation (THQ) and hazard index (HI) to assess health risks. Results obtained in this study could be important for development of dietary guidelines and risk control measures in areas surrounding non-ferrous metal smelting plants.

## 2. Materials and Methods

### 2.1. Study Area and Sample Collection

Zhuzhou city is located in the eastern side of Hunan Province and downstream of Xiang River. The city has a subtropical monsoon climate with high rainfall of 1327.6 mm and an annual average temperature of 18.8 °C [31]. This climate is suitable for the growth of a variety of high-yield grain crops in Hunan Province. Zhuzhou city is the heartland of smelting activities with abundant mineral resources and is therefore also an important industrial city.

The Zhuzhou smelter (27°52′23.06″ N, 113°05′08.40″ E), founded in 1956, is one of the most important producers and exporters of Pb and Zn metal in China. The annual production capacity of Pb and Zn products, achieved through pyrometallurgical and hydrometallurgical processes, respectively, is 650,000 tons, including 100,000 tons of Pb and 550,000 tons of Zn. The smelter also recovers copper, gold, silver, cadmium and indium, and produces sulfuric acid. Although environmental protection equipment is used during the smelting process, the surrounding environment is still threatened by the smelting activities [32].

**Figure 1:**
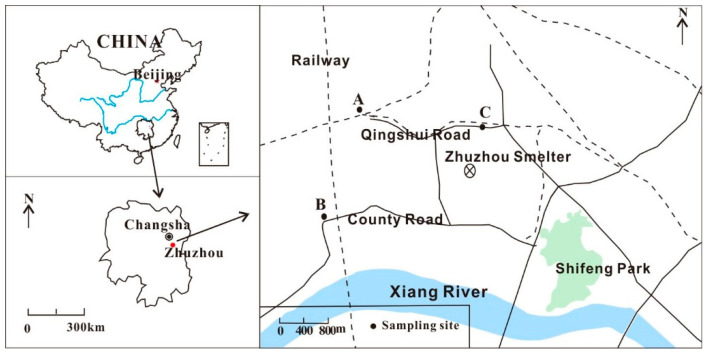
Locations of sampling sites in Zhuzhou City, Hunan.

Three sample sites (A, B and C) were selected within 3 km of the plant (Figure 1). Local residents who had been living in these areas for at least 6 months were recruited for voluntary participation in this study. Dyers and occupational exposed population were also excluded. Hair samples of 1–2 cm in length were cut with stainless steel scissors from the nape of the neck, close to the occipital region of the scalp, and then stored in plastic bags. White rice (*Oryza sativa* L.) samples were collected from the homes of the volunteers at the same time. Sources of sampled rice were recorded to determine whether the rice was cultivated in local areas or purchased from the market. Vegetable samples collected from farmlands in the residential areas were divided into leafy vegetables, including Chinese cabbage (*Brassica rapa* L. var. *chinensis* (Linnaeus) Kitamura), water spinach (*Ipomoea aquatica* Forsskal) and lettuce (*Lactuca sativa* L. var. *ramosa* Hort.) and non-leafy vegetables, including green bean (*Phaseolus vulgaris* L.) and cowpea (*Vigna unguiculata* (L.) Walp.)). A total of 115 hair samples, 69 polished rice samples and 24 vegetable samples were collected in September 2017. The rice and vegetable samples were washed with pure water, dried in an oven at 50 °C and ground to 120 mesh. The hair samples were washed with detergent, pure water and acetone, air dried and cut into sections of 1–3 mm long with stainless steel scissors for further analysis [33]. All participants signed a consent form before participating in the study. Ethics approval was obtained from the Institute of Geochemistry, Chinese Academy of Science and the Ethics Committee of Guizhou Medical University.

### 2.2. Chemical Analysis

#### 2.2.1. Heavy Metal Concentrations

Hair (50 mg), rice (100 mg) and vegetable (100 mg) samples were placed in the Teflon digestion tanks, added with 3 mL HNO_3_ and heated in an oven at 150 °C for 24 h. These solutions were cooled, filtered and diluted with HNO_3_ (2%, *v*/*v*) to analyze the concentrations of heavy metals (Cu, Zn, Cd and Pb) using an inductively coupled plasma mass spectrometer (ICP-MS; Agilent 7700, USA) [34]. Quality control was undertaken using method blanks, certified reference materials (CRMs) (NIES-13, GBW09101b, GBW10020 and GBW100359) and blind duplicates. Recoveries of CRMs averaged at 93% ± 9% for Cu, Zn, Cd and Pb. The relative standard deviations (RSDs) of replicate samples were lower than 10% and this is acceptable for trace element analysis.

#### 2.2.2. Lead (Pb) Isotope

Digested solutions were diluted with Milli-Q water to 10 μg/L of Pb in 2% HNO_3_ (*v*/*v*) and Pb isotopic compositions were measured by ICP-MS (Agilent 7900, Santa Clara, CA, USA). The lead isotopic standard (NIST SRM 981) was used for quality control and compositional correction for mass discrimination. The measured Pb compositions of ^204^Pb/^207^Pb, ^206^Pb/^207^Pb and ^208^Pb/^206^Pb in SRM 981 were 0.0645 ± 0.0003, 1.0936 ± 0.0025 and 2.1808 ± 0.0053, which were in agreement with certificated values of 0.0645, 1.0933 and 2.1681, respectively. The RSDs of replicate samples were generally lower than 0.5%.

### 2.3. Health Risk Assessment

The pathway of human heavy metal exposure includes drinking water intake, water-skin contact, air inhalation, soil intake, soil–skin contacts and food intake [30]. To study the health risk of heavy metal exposure through food consumption, the average daily dose (ADD), target hazard quotation (THQ) and hazard index (HI) were applied to calculate the potential non-carcinogenic risks [23,35].

The food intake for heavy metal exposure was calculated using Equation (1):

ADD = (C × IR × EF × ED)/(BW × AT)(1)

where ADD is the average daily dose (mg/kg/d); C is the concentration of heavy metal in rice or vegetables (mg/kg); IR is intake rate (g/d) [36] EF is exposure frequency (365 d/year); ED is exposure duration (70 years in this study); BW is body weight (kg) [31] and AT is average time (AT = ED × 365 d). Data of IR and BW are listed in Table S1.

Non-carcinogenic risk was calculated by Equations (2) and (3):

THQ = ADD/RfD(2)

HI = THQ_Cu_ + THQ_Zn_ + THQ_Cd_ + THQ_Pb_ (3)

where THQ is target hazard quotation; RfD is the reference dose (40, 300, 1 and 3.5 μg/kg/day for Cu, Zn, Cd and Pb, respectively) [37] and HI is the sum of THQ for multiple toxic substances. When HI ≤ 1, the risk is considered to be slight or negligible and when HI > 1, there is a non-carcinogenic risk.

### 2.4. Statistical Analysis

Statistical analysis was performed by SPSS19.0 for windows. The Shapiro–Wilk test was used to examine the normality of data. The characteristics of heavy metal concentrations were described by geomean. Analysis of variance (ANOVA) was conducted to investigate the differences of four heavy metals concentrations among different samples. Results of the statistical test were considered statistically significant if *p* < 0.05.

## 3. Results and Discussion

### 3.1. Heavy Metals in Rice and Vegetables

**Figure 2:**
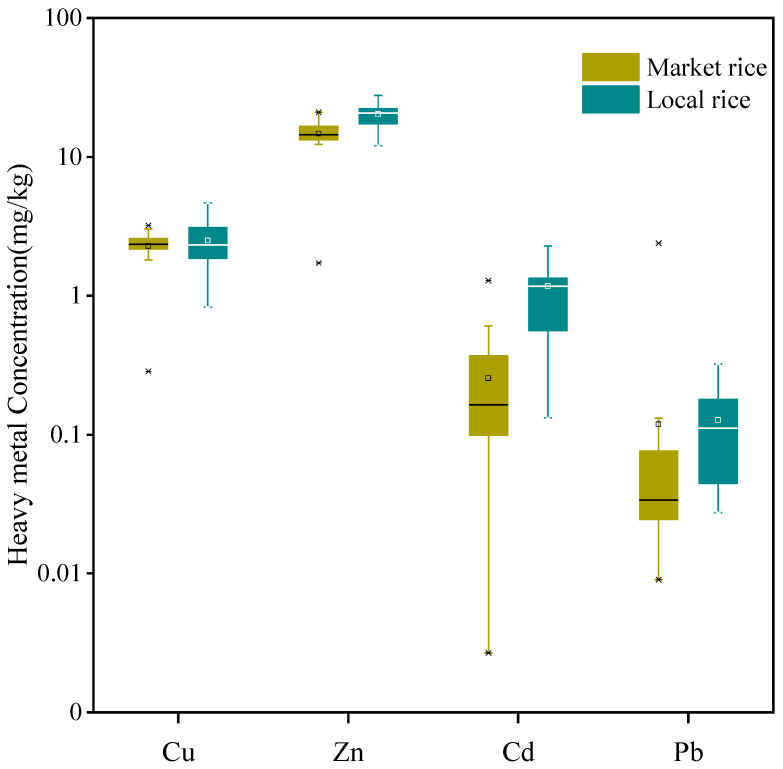
Box plots of heavy metal concentrations in rice (dry weight). Middle band, box and whiskers represent the median, 25th and 75th percentile and 5th and 95th percentile, respectively. Squares represent means, whereas ‘‘*’’ represent extreme values.

The correlation of Cu, Zn, Cd and Pb in rice and vegetables were shown in Table S2 and the concentrations of heavy metals were significantly correlated with each other. The Cu, Zn, Cd and Pb concentrations in rice samples ranged from 0.28–4.66, 1.72–31.4, <0.01–3.35 and 0.01–2.40 mg/kg, respectively. National maximum allowable concentrations of heavy metals in rice were 0.2 mg/kg for Cd and 0.2 mg/kg for Pb in China [38] and the limits were 10 mg/kg for Cu and 60 mg/kg for Zn, set by World Health Organization [39,40]. The averages of Cu and Zn concentrations in all rice samples were 2.20 ± 0.82 and 16.6 ± 4.55 mg/kg, respectively. All the concentrations of Cu and Zn in rice were below these limits. The averages concentrations of Cd and Pb in all rice samples were 0.35 ± 0.71 and 0.06 ± 0.29 mg/kg, respectively, while 70% (48/69) of the samples exceeded the national maximum allowable Cd standard (0.2 mg/kg) and 14% (10/69) of the samples exceeded the national maximum allowable Pb standard (0.2 mg/kg). The heavy metals concentrations in rice from different sites were shown in Table S3, and significant differences in Zn, Cd and Pb were found among three sites (*p* < 0.01). Significant differences in Cd and Pb concentrations were found between local rice and market rice (*p* < 0.01) (Figure 2). Local rice contained significantly higher Cd and Pb concentrations than market rice. The Cd concentrations in local rice ranged from 0.132–3.35 mg/kg, with an average of 0.913 mg/kg, while the Cd concentrations in market rice ranged from 0.003–1.29 mg/kg, with an average of 0.160 mg/kg. The portions exceeding the national limit of Cd (0.2 mg/kg) were 97% (30/31) in local rice and 47% (18/38) in market rice, respectively. The Pb concentrations in local rice ranged from 0.03–0.32 mg/kg, with an average of 0.10 mg/kg, while the Pb concentrations in market rice ranged from 0.01–2.40 mg/kg, with an average of 0.05 mg/kg. The portions exceeding the national limit (0.2 mg/kg) were 23% (7/31) in local rice and 8% (3/38) in market rice, respectively. Results in this study indicated that the rice grains grown near the smelter were seriously impacted by smelting activities and the ability of rice to enrich Cd is greater than that of Pb.

**Figure 3:**
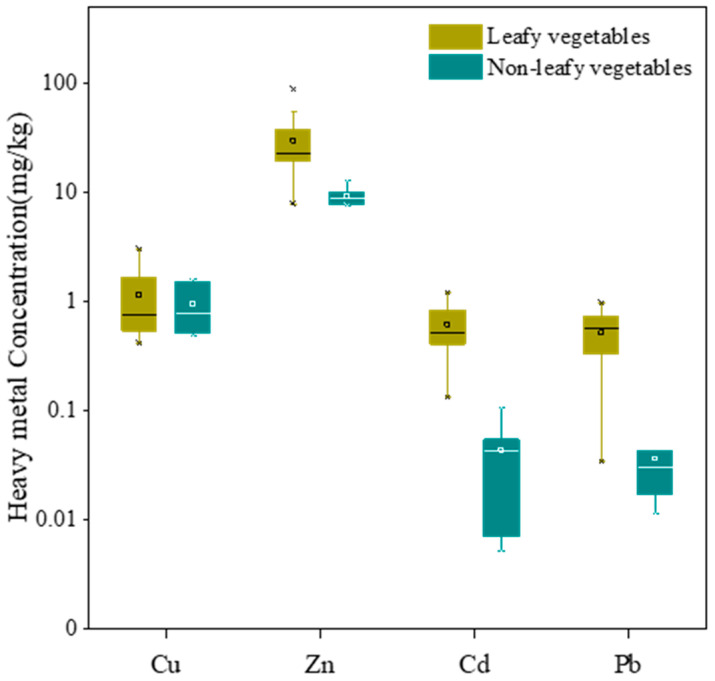
Box plots of heavy metal concentrations in vegetables (fresh weight). Middle band, box and whiskers represent the median, 25th and 75th percentile and 5th and 95th percentile, respectively. Squares represent means, whereas ‘‘*’’ represent extreme values.

The Cu, Zn, Cd and Pb concentrations in vegetable samples varied from 0.42–3.05, 7.66–89.4, 0.01–1.22 and 0.01–0.98 mg/kg, respectively. Heavy metal concentrations in leafy and non-leafy vegetables are shown in Figure 3. Significant differences in Zn, Cd and Pb concentrations were found between these two groups (*p* < 0.01), while there was no significant difference for Cu concentrations (*p* > 0.05). Geomeans of Cu, Zn, Cd and Pb concentrations in leafy vegetables were 0.92 (0.42–3.05 mg/kg), 25.4 (7.88–89.4 mg/kg), 0.54 (0.13–1.22 mg/kg) and 0.42 mg/kg (0.03–0.98 mg/kg), respectively. Geomeans of Cu, Zn, Cd and Pb concentrations in non-leafy vegetables were 0.84 (0.48–1.63 mg/kg), 9.20 (7.66–13.0 mg/kg), 0.03 (0.01–0.11 mg/kg) and 0.03 mg/kg (0.01–0.09 mg/kg), respectively. The national limits for Cd and Pb in leafy vegetables were 0.2 and 0.3 mg/kg in China [38], respectively, and the limits for Cu and Zn in leafy vegetables were 40 and 60 mg/kg as determined by the World Health Organization and Food/Agricultural Organization, respectively [41]. The national limits for Cd and Pb in non-leafy vegetables were 0.1 and 0.2 mg/kg in China [38], respectively, and the limits for Cu and Zn in non-leafy vegetables were 40 and 60 mg/kg as determined by the World Health Organization/Food and Agricultural Organization, respectively [41]. Only one sample (1/6) of non-leafy vegetables exceeded the national limit for Cd concentration. However, for the leafy vegetables, the portions of Zn, Cd and Pb concentrations exceeding national limits were 11 (2/18), 94 (17/18) and 78% (14/18), respectively. Among the vegetable samples, the Zn, Cd and Pb concentrations in leafy vegetables were obviously higher than those in non-leafy vegetables, indicating that the Zn, Cd and Pb elements became enriched in leafy vegetables.

### 3.2. Factors Impacting Heavy Metals in Hair

Human hair, a useful biomonitoring tool, has been used to evaluate the extent of heavy metal exposure in residents in contaminated industrial areas [20,42,43]. The Cu, Zn, Cd and Pb concentrations in hair samples averaged 13.1 (5.86–141 mg/kg), 202 (42.8–2180 mg/kg), 0.63 (0.05–31.4 mg/kg) and 13.6 mg/kg (1.12–260 mg/kg), respectively. The portions of Cu, Zn, Cd and Pb concentrations exceeding the TERCC reference values were 13 (15/115), 36 (41/115), 50% (58/115) and 57% (66/115), respectively, revealing health risks of heavy metals exposure for the local residents.

The comparison of heavy metal concentrations in hair with those in other studies is shown in Table S4. Hair Cu concentrations in this study were significantly higher than those in general industrial areas [22], mining areas [23], an urban area in Spain [44] and a surgical instrument manufacturing industry area in Pakistan [45], but were much lower than those from an e-waste recycling area [22], an urban area in Sweden, a non-industrial area in Italy [46] and a tailings dump area in Zambia [47]. Hair Zn concentrations in this study were much higher than those in other studies. Hair Cd concentrations in this study were comparable with those from the e-waste recycling area in China [22] and urban area in Spain [39] but were much lower than those from the surgical instrument manufacturing industry area in Pakistan [45]. Hair Cd concentrations in this study were much higher than those in general industrial areas [22], mining areas [23] in China, a tailings dump area in Zambia [47], an urban area in Sweden [48] and a non-industrial area in Italy [46]. Hair Pb concentrations in this study were higher than those in most of the above comparison areas but were lower than those in the e-waste recycling area [22] and the urban area in Spain [44]. Overall, the population surrounding the Zn smelter was subject to health risks associated with heavy metal exposure, and the exposure levels were equivalent to those in typical industrial areas.

**Figure 4:**
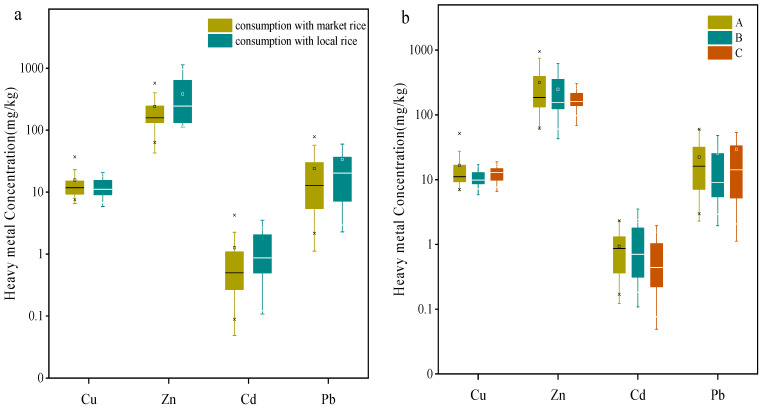

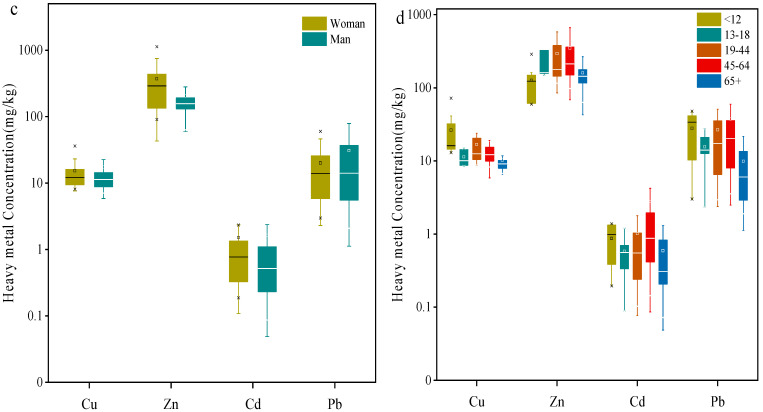
Heavy metal concentrations in human hair of as a function of (**a**) rice sources; (**b**) distance to smelter (A, B, C represented three sampling sites); (**c**) genders and (**d**) age. Middle band, box and whiskers represent the median, 25th and 75th percentile and 5th and 95th percentile, respectively. Squares represent means, whereas ‘‘*’’ represent extreme values.

Previous studies showed that food consumption was the major pathway of heavy metals exposure for human in polluted areas except occupational exposure [10]. Since rice is the staple food in south China, rice intake is the main source of human heavy metal exposure, especially for Cd. Hair heavy metals concentrations in the population with different rice consumptions are shown in Figure 4a. Significant differences in hair Zn and Cd concentrations were found between local residents who had eaten local rice and those who had consumed market rice (*p* < 0.05), while there was no significant difference for hair Cu and Pb concentrations between the two groups (*p* > 0.05). This was consistent with the characteristics of heavy metal concentrations in rice. Although the rice Pb concentration showed significant differences between two sources (*p* < 0.01), no significant difference in hair Pb concentrations was observed between local residents who had eaten local rice and those who had consumed market rice, due to the relatively low Pb concentrations in most rice samples. These results demonstrated that dietary habits could influence levels of heavy metal exposure.

Factors including gender, age and distance away from smelter that may affect the heavy metal concentrations in hair have been addressed in previous studies [22,23,49]. These factors were also considered when investigating the characteristics of human health risks in this study. Cu, Zn, Cd and Pb concentrations in hair samples from three sampling sites around the smelter are shown in Figure 4b. No significant difference in heavy metals concentrations in hair was found among the sampling sites (*p* > 0.05). Previous studies revealed significant differences in hair Cd concentrations between the mining area and the control areas (20 km to the mining area) [23]. In this study, however, the three sampling sites were located within 3 km of the smelter, indicating no differences in hair heavy metals concentrations. Concentrations of Cu, Zn, Cd and Pb in hair samples between different genders are shown in Figure 4c. No significant differences in Cu, Cd and Pb concentrations in hair were found between the two genders (*p* > 0.05), while significant differences in hair Zn were found between the genders (*p* < 0.01). The hair Zn concentrations were much higher in women than men and this was consistent with the previous results that showed that diet was the principal source of Zn and hair Zn concentrations were gender related [49].

Heavy metals concentrations in human hair among different age groups are shown in Figure 4d. Age had significant impacts on hair Cu, Zn, Cd and Pb concentrations. Hair Cu, Cd and Pb concentrations in groups of <12 years and 45–64 years were much higher than those in other age groups. Groups of 19–44 years and 45–64 years showed higher levels of hair Zn than those in other groups. These results suggested that children and older people were more sensitive to environmental Cu, Cd and Pb exposure than people of other ages and the metabolism of heavy metals in the human body differed among the various age groups.

### 3.3. Pb Isotope Tracing

**Figure 5:**
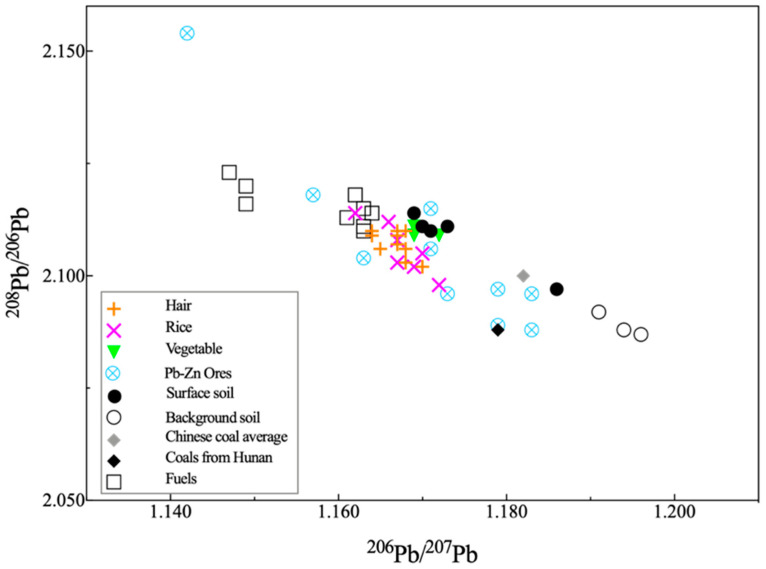
Pb isotopic compositions of rice, vegetable and human hair surrounding the smelter. The data of local coals, Chinese coals, Pb-Zn ores and fuels (gasoline and diesel) were adopted from reference [26]; surface soil and background soil from reference [50].

Ratios of Pb isotopes in the rice, vegetables and human hair were determined to identify and trace the sources of Pb exposure in the local population. Ratios of ^206^Pb/^207^Pb and ^208^Pb/^206^Pb are shown in Figure 5 and Table S5. The ^206^Pb/^207^Pb ratios ranged from 1.162–1.172 in rice samples, 1.169–1.172 in vegetables samples and 1.164–1.170 in human hair samples, respectively. The ^208^Pb/^206^Pb ratios ranged from 2.098–2.114 in rice samples, 2.109–2.111 in vegetable samples and 2.102–2.110 in human hair samples, respectively.

Smelting activity was an important source of heavy metals in the soil surrounding the smelter and the isotopic ratios of Pb in the soils (1.169–1.186 for ^206^Pb/^207^Pb and 2.097–2.114 for ^208^Pb/^206^Pb) were close to those of ores (1.142–1.183 for ^206^Pb/^207^Pb and 2.088–2.154 for ^208^Pb/^206^Pb), confirming that smelting activity was the principal contributor to Pb in the surface soils [50]. The ratios of ^206^Pb/^207^Pb and ^208^Pb/^206^Pb for rice and vegetables were comparable to those of contaminated surface soil and Pb–Zn ores, and different from those in background soil (1.191–1.196 for ^206^Pb/^207^Pb and 2.087–2.092 for ^208^Pb/^206^Pb) [50]. This suggests the clear anthropogenic influence of Pb and Zn smelting activities on these rice and vegetables. Moreover, the two main sources of Pb in edible parts of Chinese cabbage were derived from atmospheric absorption through leaf stomata and soil absorption by plant roots [51]. The isotopic ratios of Pb in rice samples in this study were close to the Pb isotopic value released by fuel (diesel and gasoline) combustion (1.147–1.164 for ^206^Pb/^207^Pb and 2.110–2.123 for ^208^Pb/^206^Pb) [26], indicating that the use of fuels may affect the Pb isotopes in plants through atmospheric absorption.

In this study, results showed that Pb in vegetables mainly originated from root uptake from the soil and Pb in rice could have originated from the mixtures of leaf atmospheric absorption and root absorption from soil. Pb isotopes in human hair were comparable to those in the rice and were slightly lower than those of the vegetables, indicating that human Pb exposure likely mainly originated from the daily intake of rice.

### 3.4. Health Risk Assessment by Rice and Vegetables

**Figure 6:**
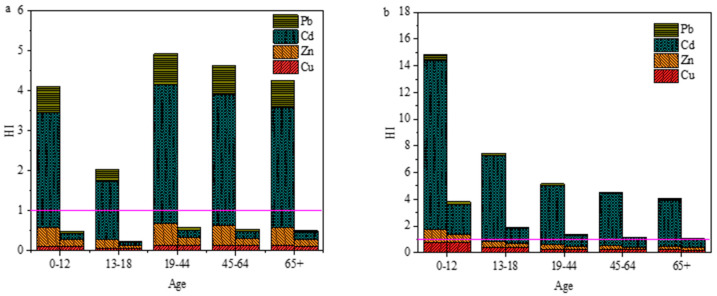
Hazard index (HI) in different scenarios of consumption. (**a**) vegetables (left columns represent leafy vegetables and right columns represent non-leafy vegetables); (**b**) rice (left columns represent local rice and right columns represent market rice).

Exposure of human to high concentrations of heavy metals such as Cu, Zn, Cd and Pb would pose health risks. Non-carcinogenic risks for local residents were assessed through the dietary pathway in this study. Generally, rice and vegetables are the most significant sources of heavy metals exposure in daily human life, especially in south China [9,29,52]. We calculated the non-carcinogenic risks of selected heavy metals for local residents from rice and vegetable ingestion among different age groups (Figure 6 and Table S6).

In Figure 6, THQ values were calculated for different scenarios under the assumption that residents only ingest one type of vegetables or rice. In Figure 6a, the THQ values for leafy and non-leafy vegetables were in the same order: Cd > Pb > Zn > Cu. For non-leafy vegetables, the THQ values of Cu, Zn, Cd and Pb were all <1 and this did not exceed the guideline value. Moreover, the HI values of non-leafy vegetables were also <1 and the health risks from non-leafy vegetable consumption were considered slight or negligible. For leafy vegetables, although the THQ values of Cu, Zn and Pb were <1, the THQ values of Cd were 1.42–3.46 times higher than the threshold value. This showed that the health risks of heavy metal exposure for the local population mainly came from Cd and Pb exposure through leafy vegetable consumption. In addition, significant differences of HI values among different age groups for vegetable ingestion were found. HI values for different age groups followed the order: 19–44 > 45–64 > 65+ > 0–12 > 13–18 for both leafy and non-leafy vegetables consumption. HI values of leafy vegetables consumption for all age groups were >1, thereby posing health risks to local residents, while HI values of non-leafy vegetables consumption for all age groups were less than one, which was considered as safe.

In Figure 6b, the THQ values for local rice and market rice followed the following order: Cd > Zn > Cu > Pb and Cd > Cu > Zn > Pb, respectively. For the five age groups, regardless of the type of rice consumed, the THQs of Cu, Zn and Pb were all below the threshold value of one. However, THQs of Cd in five age groups with local rice consumption and age groups 0–12 and 13–18 with market rice consumption were higher than the threshold value of one. Therefore, rice intake was considered the predominant health risk of Cd exposure for local residents around the smelter. Although the THQ values of Cu, Zn, Cd and Pb for the age groups of 19–44, 45–64 and 65+ with market rice consumption were lower than one, the HI values in the three groups were higher than one. Therefore, rice consumption from different sources would cause different levels of exposure to heavy metals. The HI values of market rice were significantly lower than those of local rice. Rice occupies a dominant position in the dietary structure of residents in south China and heavy metal exposure through consumption of wheat products is much lower than that through rice consumption [9]. To reduce the health risks of heavy metals exposure through local rice, consumption of commercial rice and dietary structure adjustment are needed for local residents. Control of anthropogenic heavy metal emissions and soil remediation are also needed to reduce heavy metal bioaccumulation in agricultural crops and associated health risks in the local population.

## 4. Conclusions

Heavy metal contaminations in rice and vegetables surrounding a Pb/Zn smelter were evaluated and the health risk assessments using THQ and HI through rice and vegetables intake were conducted in this study. Heavy metals concentrations in locally cultivated rice and leafy vegetables were significantly elevated than those in market rice and non-leafy vegetables. The sources of rice were an important factor affecting the distribution of Zn and Cd in human hair. Children and the elderly were more sensitive to environmental Cu, Cd and Pb exposure. Hair Zn was mainly originated from dietary intake and was gender related. Pb isotopic compositions in rice, vegetable and hair samples demonstrated that rice consumption was the main source of Pb exposure in local residents. Locally cultivated rice and leafy vegetables posed higher non-carcinogenic risks to the local residents than market rice and non-leafy vegetables. Therefore, control of the emission of heavy metals from the smelter and implementation of soil remediation in the surrounding area are urgently needed. Furthermore, based on the results from this study, adjustment of crop planting structures and daily dietary structures of local residents are needed to reduce heavy metal exposure risks through local rice and leafy vegetable ingestion in this area.

## Data Availability

The data presented in this study are available on reasonable request from the corresponding authors.

## Supplementary Materials

The following are available online at https://www.mdpi.com/article/10.3390/ijerph182312631/s1, Table S1: Parameters of intake rate (IR) of rice and vegetable and body weight (BW) among different age groups; Table S2: correlation coefficients between heavy metal concentrations in rice, vegetables and hair; Table S3: heavy metal concentrations in rice from difference sites (mg/kg, DW); Table S4: Comparison of heavy metals concentrations in hair from different living areas (mg/kg); Table S5: Pb isotopic compositions in rice, vegetables, human hair, soil, coals and fuels; Table S6: THQ and HI values of heavy metals for local residents via rice or vegetable consumption.
