# Supplementary material for: Heavy Metal in Rice and Vegetable and Human Exposure near a Large Pb/Zn Smelter in Central China
Source: Int J Environ Res Public Health. 2021 Nov 30;18(23):12631. doi: 10.3390/ijerph182312631 (PMC8657013; doi:10.3390/ijerph182312631)
Supplement: Supplementary file 1 [file ijerph-18-12631-s001.zip › ijerph-1471782-supplementary.pdf]

Supplementary Materials to

Heavy metal in rice and vegetable and human exposure near a large Pb/Zn smelter in Central China

Yanxin Hu[1], Chuan Wang[1,2], Zhengcheng Song[1], Min Chen[3], Li Ding[3], Xingyu Liang[1,2], Xiangyang Bi[4], Zhonggen Li[5], Ping Li[1,*], Wei Zheng[6,*]

[1]State Key Laboratory of Environmental Geochemistry, Institute of Geochemistry, Chinese Academy of Sciences, Guiyang 550081, China

[2]College of Earth and Planetary Sciences, University of Chinese Academy of Sciences, Beijing 100049, China

[3]School of Public Health, Guizhou Medical University, Guiyang, 550025, China

[4]Hubei Key Laboratory of Critical Zone Evolution, School of Earth Sciences, China University of Geosciences, Wuhan 430074, China

[5]School of Resources and Environment, Zunyi Normal College, Zunyi 563006, China

[6]Institute of Atmospheric Environment, Chinese Academy of Environmental Planning, Beijing 100012, China

*Correspondence: Ping Li, Email: liping@mail.gyig.ac.cn (P.L.); Wei Zheng, Email: zhengwei@caep.org.cn (W.Z.)

Table S1. Parameters of intake rate (IR) of rice and vegetable and body weight (BW) among different age groups

age	IR of rice (g)	IR of vegetable (g)	BW (kg)	References
0-12	342.6	131.4	24.6	[1]
13-18	358.29	135.5	51	
18-44	298.9	394	61.5	
45-64	267.97	378.78	62.9	
65+	223.95	326.05	59	

Table S2 correlation coefficients between heavy metal concentrations in rice, vegetables and hair

Rice	Cu	Zn	Cd	Pb	Vegetables	Cu	Zn	Cd	Pb	Hair	Cu	Zn	Cd	Pb
Cu	1.000				Cu	1.000				Cu	1.000			
Zn	0.192*	1.000			Zn	0.014	1.000			Zn	.208**	1.000		
Cd	0.178*	0.639**	1.000		Cd	0.123	0.645**	1.000		Cd	.230**	.220**	1.000	
Pb	0.047	0.410**	0.425**	1.000	Pb	0.203	0.507**	0.674**	1.000	Pb	.315**	.170**	.673**	1.000

* $p<0.05$, ** $p<0.01$

Table S3 heavy metal concentrations in rice from difference sites (Geomean ± SD, mg/kg, DW)

Site	n	Cu	Zn	Cd	Pb
A	24	1.97±0.80	17.3±4.47	0.50±0.74	0.10±0.47
B	28	2.32±0.99	17.3±5.08	0.60±0.76	0.06±0.07
C	17	2.33±0.38	14.0±1.57	0.08±0.18	0.04±0.06

Table S4. Comparison of heavy metals concentrations in hair from different areas (mg/kg)

Region	Description	N	Values	Cu	Zn	Cd	Pb	References
Zhuzhou, China	lead/zinc smelting area	115	Range	5.86-140.71	42.78-2179.41	0.05-31.35	1.12-259.71	This study
			Mean	13.08	202.10	0.63	13.58	
			SD	19.10	294.11	4.20	38.27	
Taizhou, China	e-waste recycling area	139	Range	10.85-537	-	0.01-13.70	1.93-730	[2]
			Mean	39.80	-	0.52	49.50	
			SD	1.94	-	3.11	2.93	
Ningbo, China	general industrial area	10	Range	5.27-14.0	-	0.18-0.33	1.09-8.77	[2]
			Mean	9.93	-	0.23	2.53	
			SD	1.29	-	1.26	2.12	
Shaoxing, China	general industrial area	10	Range	8.49-11.8	-	0.12-0.34	3.50-15.9	[2]
			Mean	10.10	-	0.21	6.61	
			SD	1.24	-	1.38	1.70	
Yunnan, China	mining area	60	Range	4.81-11.3	59-360	0.021-1.1	2.49-12.60	[3]
			Mean	8.70	172.00	0.22	9.04	
			SD	6.20	58.00	0.28	2.28	
	control area	26	Range	5.51-15.2	89.1-238	0.01-0.28	2.77-6.64	
			Mean	9.82	163.00	0.12	5.04	
			SD	2.20	37.00	0.09	3.11	
Spain	urban area	22	Range	2.21-38.08	5.81-78.67	0.03-0.33	3.03-290.46	[4]
			Mean	10.78	34.51	0.11	41.32	
			SD	6.44	16.50	0.06	47.59	

Sweden	urban area	114	Range	8.5-96	68-198	0.01-0.356	0.22-7.26	[5]
			Mean	25.00	142.00	0.06	0.96	
			SD	21.00	29.00	0.06	0.85	
Italy	non-industrial area	132	Range	9.1-59.7	96.86-329.19	0.0004-0.16	0.28-3.03	[6]
			Mean	22.87	189.20	0.11	1.01	
			SD	12.39	59.30	0.12	0.76	
Zambia	tailings dump area	-	Range	3.6-143	39-192	0.02-2.14	0.3-15.8	[7]
			Mean	38.00	137.00	0.30	4.30	
			SD	6.90	21.10	0.02	1.95	
Pakistan	surgical instrument manufacturing industry	6	Range	0.43-0.53	1.08-1.98	0.15-2.03	0.14-0.68	[8]
			Mean	0.49	1.52	1.40	0.39	
			SD	0.05	0.37	0.86	0.25	

Table S5. Pb isotopic compositions in rice, vegetables, human hair, soil, coals and fuels.

Sample	$^{206}Pb/^{204}Pb$	$^{206}Pb/^{207}Pb$	$^{208}Pb/^{206}Pb$	Reference
rice	16.669	1.162	2.114	This study
	16.358	1.172	2.098	
	17.886	1.170	2.105	
	17.233	1.167	2.103	
	16.646	1.169	2.102	
	17.621	1.166	2.112	
	17.704	1.167	2.108	
vegetable	18.273	1.169	2.111	This study
	18.380	1.172	2.109	
	18.148	1.169	2.110	
hair	17.965	1.168	2.103	This study
	18.017	1.164	2.109	
	17.858	1.167	2.109	
	17.925	1.165	2.106	
	18.264	1.167	2.107	
	17.439	1.164	2.110	
	18.211	1.168	2.110	
	15.454	1.168	2.106	
	17.488	1.167	2.110	
	17.949	1.170	2.102	
surface soil	18.545	1.169	2.114	[9]
	18.483	1.170	2.111	
	18.422	1.173	2.111	
	18.661	1.186	2.097	
	18.306	1.171	2.110	
background soil	18.725	1.194	2.088	[9]
	18.468	1.196	2.087	
	18.383	1.191	2.092	
Pb-Zn ores	18.458	1.171	2.115	[10]
	17.796	1.142	2.154	
	18.299	1.163	2.104	
	18.406	1.173	2.096	
	18.048	1.157	2.118	
	18.305	1.171	2.106	
	18.600	1.183	2.096	
	18.480	1.179	2.097	
	18.307	1.183	2.088	
	18.398	1.179	2.089	
Coals from Hunan province	18.286	1.179	2.088	[10]
Chinese coal average	18.544	1.188	2.100	[10]

Diesel	18.132	1.162	2.118	[10]
	18.2	1.164	2.114	
	18.212	1.163	2.115	
	18.104	1.161	2.113	
	18.133	1.163	2.110	
	18.163	1.163	2.111	
Gasoline	17.955	1.149	2.116	[10]
	17.997	1.149	2.120	
	18.034	1.147	2.123	

Table S6. THQ and HI values of heavy metals for local residents via rice or vegetable consumption

Type	Age	THQ				HI
		Cu	Zn	Cd	Pb	
Local rice	0-12	0.80	0.92	12.67	0.40	14.80
	13-18	0.41	0.47	6.39	0.20	7.47
	19-44	0.28	0.32	4.42	0.14	5.16
	45-64	0.25	0.28	3.88	0.12	4.53
	65+	0.22	0.25	3.45	0.11	4.03
Market rice	0-12	0.73	0.65	2.23	0.20	3.82
	13-18	0.37	0.33	1.12	0.10	1.92
	19-44	0.26	0.23	0.78	0.07	1.33
	45-64	0.22	0.20	0.68	0.06	1.17
	65+	0.20	0.18	0.61	0.05	1.04
Leafy vegetable	0-12	0.12	0.45	2.88	0.64	4.10
	13-18	0.06	0.22	1.43	0.32	2.04
	19-44	0.15	0.54	3.46	0.77	4.92
	45-64	0.14	0.51	3.25	0.72	4.62
	65+	0.13	0.47	2.98	0.66	4.24
Non-leafy vegetable	0-12	0.11	0.16	0.16	0.05	0.48
	13-18	0.06	0.08	0.08	0.02	0.24
	19-44	0.13	0.20	0.19	0.05	0.58
	45-64	0.13	0.18	0.18	0.05	0.54
	65+	0.12	0.17	0.17	0.05	0.50

References

1. Duan, X. Exposure Factors Handbook of Chinese Population; China Environmental Science Press: Beijing,China, 2013.

2. Wang, T., Fu, J., Wang, Y., Liao, C., Tao, Y., Jiang, G., 2009. Use of scalp hair as indicator of human exposure to heavy metals in an electronic waste recycling area. Environ Pollut 157, 2445-2451.

3. Wang, Y., Wang, R., Fan, L., Chen, T., Bai, Y., Yu, Q., Liu, Y., 2017. Assessment of multiple exposure to chemical elements and health risks among residents near Huodehong lead-zinc mining area in Yunnan, Southwest China. Chemosphere 174, 613-627.

4. Peña-Fernández, A., González-Muñoz, M.J., Lobo-Bedmar, M.C., 2014. "Reference values" of trace elements in the hair of a sample group of Spanish children (aged 6-9 years) - are urban topsoils a source of contamination? Environ Toxicol Pharmacol 38, 141-152.

5. Rodushkina Ilia and Axelsson M.D., 2000. Application of double focusing sector field ICP-MS for multi elemental characterization of human hair and nails. Part II. A study of the inhabitants of northern Sweden. Sci. Total. Environ. 262, 21-36.

6. Dongarra, G., Lombardo, M., Tamburo, E., Varrica, D., Cibella, F., Cuttitta, G., 2011. Concentration and reference interval of trace elements in human hair from students living in Palermo, Sicily (Italy). Environ Toxicol Pharmacol 32, 27-34.

7. Nakaona, L., Maseka, K.K., Hamilton, E.M., Watts, M.J., 2019. Using human hair and nails as biomarkers to assess exposure of potentially harmful elements to populations living near mine waste dumps. Environ Geochem Health.

8. Junaid, M., Malik, R.N., Pei, D.S., 2017. Health hazards of child labor in the leather products and surgical instrument manufacturing industries of Sialkot, Pakistan. Environ Pollut. 226, 198-211.

9. Liang, X., Wang, C., Song, Z., Yang, S., Bi, X., Li, Z., Li, P., 2021. Soil metal(loid)s pollution around a lead/zinc smelter and source apportionment using isotope fingerprints and receptor models, Appl. Geochem.

10. Bi, X.Y., Li, Z.G., Wang, S.X., Zhang, L., Xu, R., Liu, J.L., Yang, H.M., Guo, M.Z., 2017. Lead Isotopic Compositions of Selected Coals, Pb/Zn Ores and Fuels in China and the Application for Source Tracing. Environ Sci Technol 51, 13502-13508.
